# Affinity-Purified Respiratory Syncytial Virus Antibodies from Intravenous Immunoglobulin Exert Potent Antibody-Dependent Cellular Cytotoxicity

**DOI:** 10.1371/journal.pone.0069390

**Published:** 2013-07-19

**Authors:** Nimesh Gupta, Jerome LeGoff, Soulaima Chamat, Severine Mercier-Delarue, Olivier Touzelet, Ultan F. Power, Michel D. Kazatchkine, Francois Simon, Sebastien Lacroix-Desmazes, Jagadeesh Bayry, Srinivas V. Kaveri

**Affiliations:** 1 Institut National de la Santé et de la Recherche Médicale, Unité 872, Paris, France; 2 Centre de recherche des Cordeliers, Equipe 16-Immunopathology and Therapeutic immunointervention, Paris, France; 3 Université Pierre et Marie Curie, Université Paris Descartes, UMR S 872, Paris, France; 4 University Paris Diderot, Pres Sorbone Paris Cité, Paris, France; 5 Microbiology laboratory, Hôpital Saint-Louis, Assistance Publique-Hôpitaux de Paris, France; 6 Laboratory of Immunology, Faculty of Public Health, Lebanese University, Fanar, Lebanon; 7 Centre for Infection and Immunity, School of Medicine, Dentistry, and Biomedical Sciences, Queens University Belfast, Belfast, United Kingdom; 8 United Nation Secretary General Special Envoy on HIV/AIDS in Eastern Europe and Central Asia, Geneva, Switzerland; 9 International Associated Laboratory, Institut National de la Santé et de la Recherche Médicale-France and Indian council of Medical Research, Mumbai, India; University Paris Sud, France

## Abstract

Mixed infections are one of the major therapeutic challenges, as the current strategies have had limited success. One of the most common and widespread conditions of mixed infection is respiratory syncytial virus-mediated pathology of the respiratory tract in children. There is a dire need for the development of novel therapeutic approaches during mixed infections. Therapeutic intravenous immunoglobulin preparations, obtained from plasma pools of healthy donors have been used in immune deficiencies. This study was thus designed to characterize the functional efficacy of RSV-specific antibodies in IVIg. To explore the functional ability of these affinity-purified RSV-specific antibodies, the antibody-dependent and complement dependent cytotoxicity was determined using peripheral cells of healthy donors. This study demonstrates the existence of highly potent RSV-specific antibodies in IVIg preparations and provides the basis for the use of IVIg as broad-spectrum protective shield to RSV-infected children during mixed infections.

## Introduction

The Respiratory syncytial virus (RSV) is a common and widespread respiratory tract infectious agent. RSV usually induces a benign infection in immunocompetent individuals, [1] but is the most important cause of bronchiolitis and pneumonia in infants and young children [2], [3]. Currently, there is no effective vaccine available to prevent RSV infection. Although anti-viral drugs are proposed to treat RSV infections, their efficacy in children is debatable. RespiGam®, a preparation of pooled normal immunoglobulins that contains high titer antibodies against RSV was utilized as an alternative immunoprophylactic approach [4]. The emergence of highly specific monoclonal antibodies such as Palivizumab and Motavizumab, led to the withdrawal of RespiGam® from the market. However, the cost and the agent-specific nature of the monoclonal antibodies warrant the search for a safer and broad-spectrum immunoprophylactic alternative.

Intravenous immunoglobulin G (IVIg) is a preparation obtained from plasma pools of several thousand healthy blood donors and is used to treat various autoimmune, infectious, and idiopathic diseases [5], [6]. The content of antibodies against microbial antigens is influenced by the endemic nature of the pathogens and vaccination history of the population. Several studies have documented the existence of antibodies against pathogens including West Nile Virus, hepatitis B virus, cytomegalovirus and Plasmodium [7]–[12]. The current study was thus designed to determine the presence of RSV-specific antibodies and their functional potency after antigen-specific purification in various commercially available IVIg preparations.

## Materials and Methods

### Ethics Statement

Buffy bags from healthy donors were obtained after written consent following the approval by ethical committees of Institut national de la santé et de la recherche médicale (INSERM) and Etablissement Français du Sang (EFS), Paris, France; convention 12/EFS/079.

### Virus and Cells

RSV A strain Long was employed throughout this study and was a kind gift from Dr. Sabine Riffault (UR892, INRA, Jouy-en-Josas, France). The Human HEp-2 cells were obtained from the European cell culture collection and maintained in minimum essential medium (MEM) with Earle’s salts supplemented with 2 mM L-glutamine and 10% fetal bovine serum. Human peripheral blood mononuclear cells (PBMC) were isolated from whole blood of healthy donors by centrifugation on a Ficoll-Paque layer (PAA, Velizy-Villacoublay). PBMCs were depleted of CD4^+^ T cells by positive selection using CD4 microbeads (Miltenyi Biotec).

### Purification of Anti-RSV Antibodies from Intravenous Immunoglobulin

Recombinant RSV G protein (Sino Biological Inc.) was coupled to CNBr-activated CH Sepharose 4B (Pharmacia) according to the manufacturer’s instructions. IVIg was incubated with the affinity matrix overnight at 4°C. After washing the column with PBS until the effluent was protein-free, the bound fraction was eluted with a 0.2M glycine-HCl buffer pH 2.8 and the eluate was immediately neutralized with 4M Tris. The eluate was dialyzed against PBS and concentrated using Amicon Ultra centrifugal filters (Millipore).

### ELISA

MaxiSorp® microtiter plates (Nunc, Denmark) were coated with Recombinant G protein of RSV (2 µg/ml) in PBS by incubating for 2 h at room temperature (RT). The unbound sites were blocked using 0.25% Tween-20 in PBS for 2 h at RT. The plates were incubated with serial dilutions of different preparation of IVIg or affinity purified anti-RSV G antibodies (diluted in 0.05% Tween-20-PBS) for 2 h at RT. The plates were washed and incubated with horseradish peroxidase conjugated Goat anti-human IgG antibodies (Southern Biotech). Bound antibodies were revealed and the optical density was determined at 490 nm using an Emax ELISA reader (Molecular Devices, Menlo Park, CA).

### Antibody-dependent Cellular Cytotoxicity (ADCC)

The ADCC activity of the affinity purified anti-RSV G antibodies, anti-RSV F monoclonal antibody (MAb) (Palivizumab) and IVIg was assessed using CytoTox 96 non-radioactive cytotoxicity assay kit (Promega, France). The PBMCs (effector cells) were resuspended in X-VIVO 15 medium (Lonza, Switzerland) and incubated overnight at 37°C in the presence of 100 ng/ml recombinant human IL-2 (Miltenyi Biotec, France). HEp-2 cells (target cells) were infected with RSV at 0.5 MOI for 24 h. The cells were washed and resuspended in assay buffer {RPMI-1640 (Lonza, Switzerland) supplemented with 5% FBS (Hyclone, UK), 15 mM HEPES (Invitrogen, France)} at a density of 2×10^5^ cells/ml. The HEp-2 cells were distributed into round-bottomed 96-well plates (50 µl/well) along with various concentrations of IVIg, affinity purified anti-RSV-G antibodies and anti-RSV F MAb at 50 µl/well in assay buffer (without FBS) and the mixtures were incubated at 37°C for 30 minutes. The IL-2 stimulated and non-adherent PBMCs were then harvested and resuspened in the assay buffer at the density of 5×10^6^ cells/ml and added at 100 µl/well to the assay plates (effector: target ratio of 50∶1). The assay controls were prepared as per the protocol of cytotoxicity assay kit. The plates were centrifuged at 250×g for 4 minutes and incubated at 37°C for 4 h. For target cell maximum LDH release control, the lysis solution was added to the corresponding wells 45 min prior to the completion of incubation and the plates were centrifuged at 250×g for 4 minutes. After complete incubation, 50 µl of supernatant from each well was transferred to flat-bottom MaxiSorp 96-well plates. 50 µl of reconstituted substrate mix was then added to all wells and plates were incubated in the dark at RT for 30 min. 50 µl of stop solution was added to all wells and LDH release was quantified by measuring the absorbance at 490 nm. The experiment was performed in triplicates. The culture medium background absorbance was subtracted from all test or control absorbance values and the percentage of cytotoxicity was calculated using the following formula:
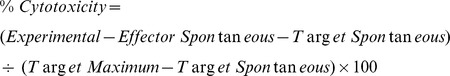



*Experimental* corresponds to the absorbance obtained in assay conditions including both target cells and effector cells with serially diluted antibodies. *Effector spontaneous* corresponds to the absorbance obtained in the presence of PBMCs alone, *Target spontaneous* corresponds to the absorbance obtained in the presence of HEp-2 cells alone and *Target maximum* corresponds to the absorbance obtained in the presence of detergent-lysed HEp-2 cells.

### Complement-dependent Cytotoxicity (CDC)

The CDC activity of the affinity purified anti-RSV G antibodies, anti-RSV F MAb and IVIg was assessed using CytoTox 96 non-radioactive cytotoxicity assay kit (Promega, France) with some modifications. Briefly, HEp-2 cells were infected with RSV at 0.5 MOI for 24 h followed by washing and resuspended in assay buffer (see above) at a density of 2×10^5^ cells/ml. The HEp-2 cells were distributed in round-bottomed 96-well plates (50 µl/well) along with various concentrations of IVIg, affinity purified anti-RSV-G antibodies and anti-RSV F antibodies at 50 µl/well in assay buffer (without FBS) and the mixtures were incubated at 37°C for 30 min. Rabbit complement sera (Sigma) was diluted in RPMI-1640 (1∶12) and added at 100 µl/well to the assay plates followed by incubation for 90 min at 37°C. The assay controls were prepared as per the manufacturer’s instructions and the culture medium with rabbit complement sera was used as an additional assay control to correct background absorbance. The percentage of cytotoxicity was determined using following formula:
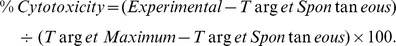



### Statistical Analysis

The differences between groups were compared using Two-Way ANOVA followed by Bonferroni test, utilyzing GraphPad Prism Software (San Diego California USA). Data were presented as mean ± standard error of values obtained in three independent experiments.

## Results and Discussion

Several studies have proposed the crucial role of RSV G protein in RSV-mediated pathology and therapeutic as well as prophylactic targeting of this glycoprotein has been anticipated to be beneficial [13]–[18]. In the present study, we thus focused on the antibodies against this important protein of RSV [19], [20].

**Figure 1:**
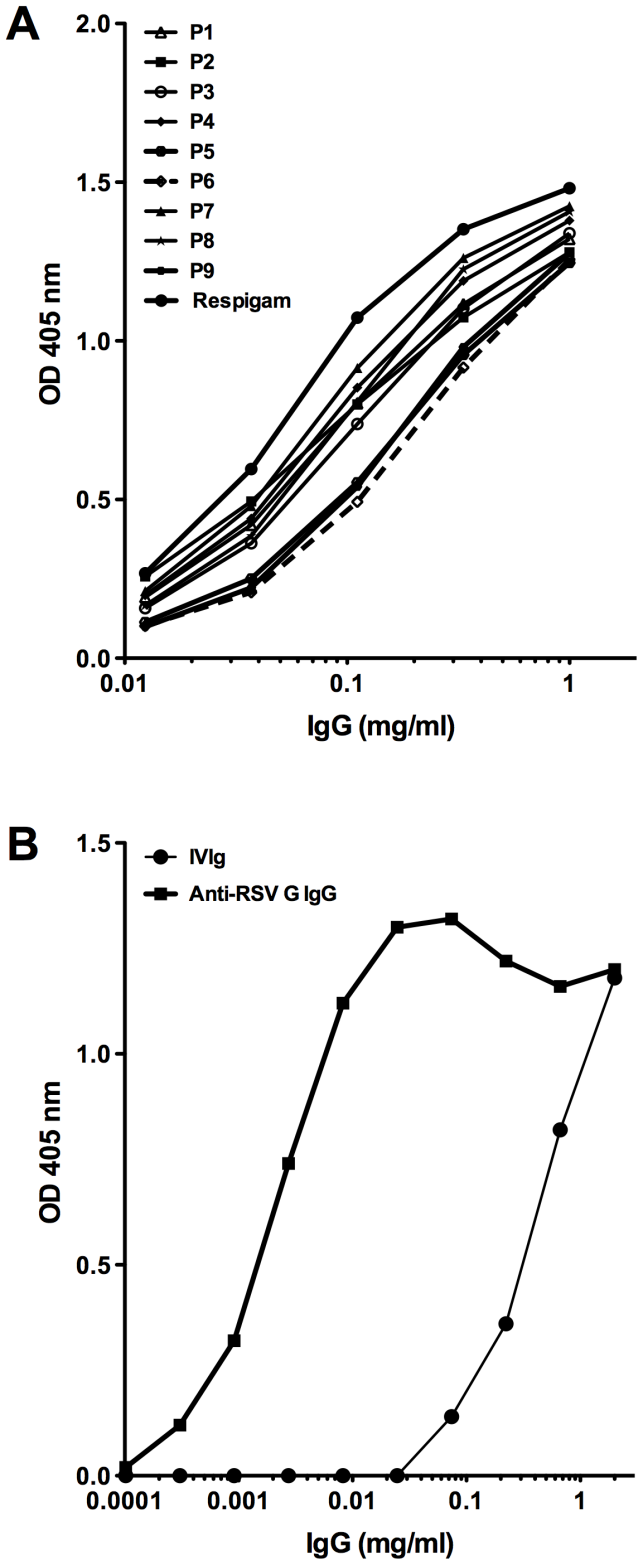
Reactivity of native IVIg and purified anti-RSV G antibodies. **A)** Reactivity of various IVIg preparations (labeled P1 to P9) with the recombinant G protein of RSV. **B)** Reactivity of IVIg (line with circle) and affinity-purified anti-RSV IgG (line with square) with the recombinant G protein of RSV.

Ten different commercial IVIg preparations including RespiGam® were analyzed for their capacity to bind to a recombinant G protein of RSV. We observed that all tested IVIg preparations recognize recombinant G protein of RSV, but these preparations have varying proportion of the specific antibodies (Figure 1A). To characterize the RSV G-reactive antibodies, we affinity purified anti-RSV G antibodies from one of the IVIg preparations, using recombinant RSV G protein coupled to CNBr-activated CH Sepharose 4B. We observed that approximately 0.15% of the loaded IVIg bound to the affinity column. This affinity-purified anti-RSV IgG recognized RSV G protein at 100-fold lower concentrations than the unfractionated total IVIg (Figure 1B).

**Figure 2:**
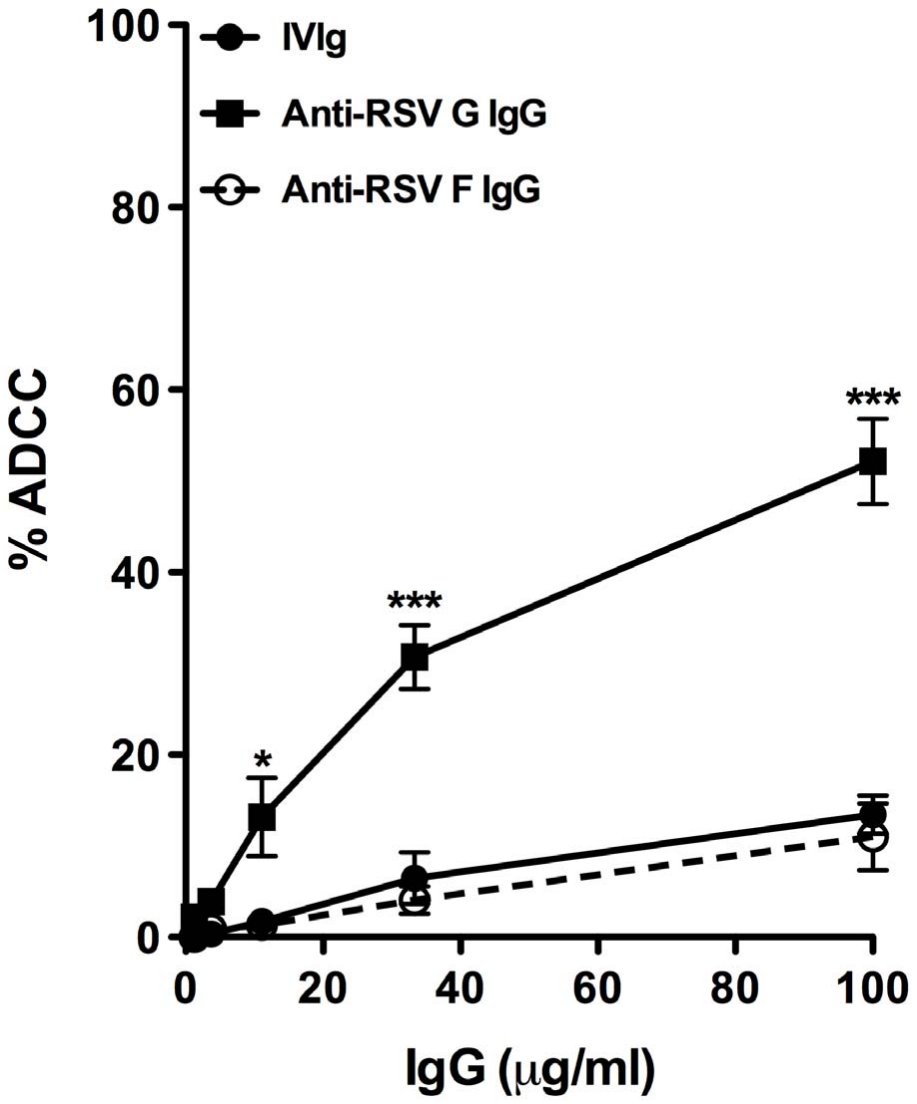
Antibody-dependent cell-mediated cytotoxicity (ADCC) activity. The RSV infected Hep-2 cells were used as target cells and PBMCs were used as effector cells. The cells were incubated with various concentrations of IVIg (line with circle), anti-RSV G IgG (line with square) and anti-RSV F IgG (line with blank circle). The assay was performed at the ratio of 50∶1 (Effector: Target) in triplicates. Data were analyzed by two-way ANOVA followed by Bonferroni test for comparison between IVIg, anti-RSV G IgG and anti-RSV F IgG treated cells. Results are mean ± standard error of the mean of values obtained with PBMCs (effector cells) from 6 individual donors in three independent experiments. ***P<0.001, and *P<0.05.

The functional potency of affinity-purified anti-RSV antibodies was examined by their ability to mediate antibody-dependent cytotoxicity of HEp-2 cells infected with RSV [21]. Results show that affinity-purified anti-RSV G antibodies mediate a potent ADCC towards RSV-infected HEp-2 cells in a dose-dependent manner (52.17%±4.6 at 100 µg/ml; 30.69%±3.5 at 33.33 µg/ml; 13.16±4.2 at 11.1 µg/ml; 3.9%±1.05 at 3.7 µg/ml and 2.19%±0.5 at 1.2 µg/ml). The unfractionated total IVIg also showed ADCC towards RSV-infected HEp-2 cells at higher concentrations (13.43%±2 at 100 µg/ml; 6.48%±2.8 at 33.33 µg/ml; 1.73±1.23 at 11.1 µg/ml; 0.35%±0.35 at 3.7 µg/ml and 0%±0.5 at 1.2 µg/ml). The ADCC observed in the presence of anti-RSV F MAb (11%±3.6 at 100 µg/ml; 4%±1.4 at 33.33 µg/ml; 1.3±0.74 at 11.1 µg/ml; 0.89%±0.58 at 3.7 µg/ml and 0%±0.5 at 1.2 µg/ml) was even lower than the ADCC exerted by unfractionated IVIg (Figure 2). However, affinity-purified anti-RSV G antibodies were 4 to 8-fold more potent than unfractionated total IVIg or anti-RSV F MAb in attaining significant lysis (Figure 2). The possible involvement of ADCC by anti-RSV-G antibodies in the virus clearance has been proposed [17], [18] and our results confirms that anti-RSV G antibodies purified from the IVIg preparation have the potential to exert ADCC. The low ADCC by anti-RSV F MAb in our experiments suggest that ADCC is not an important effector function of this antibody [22].

**Figure 3:**
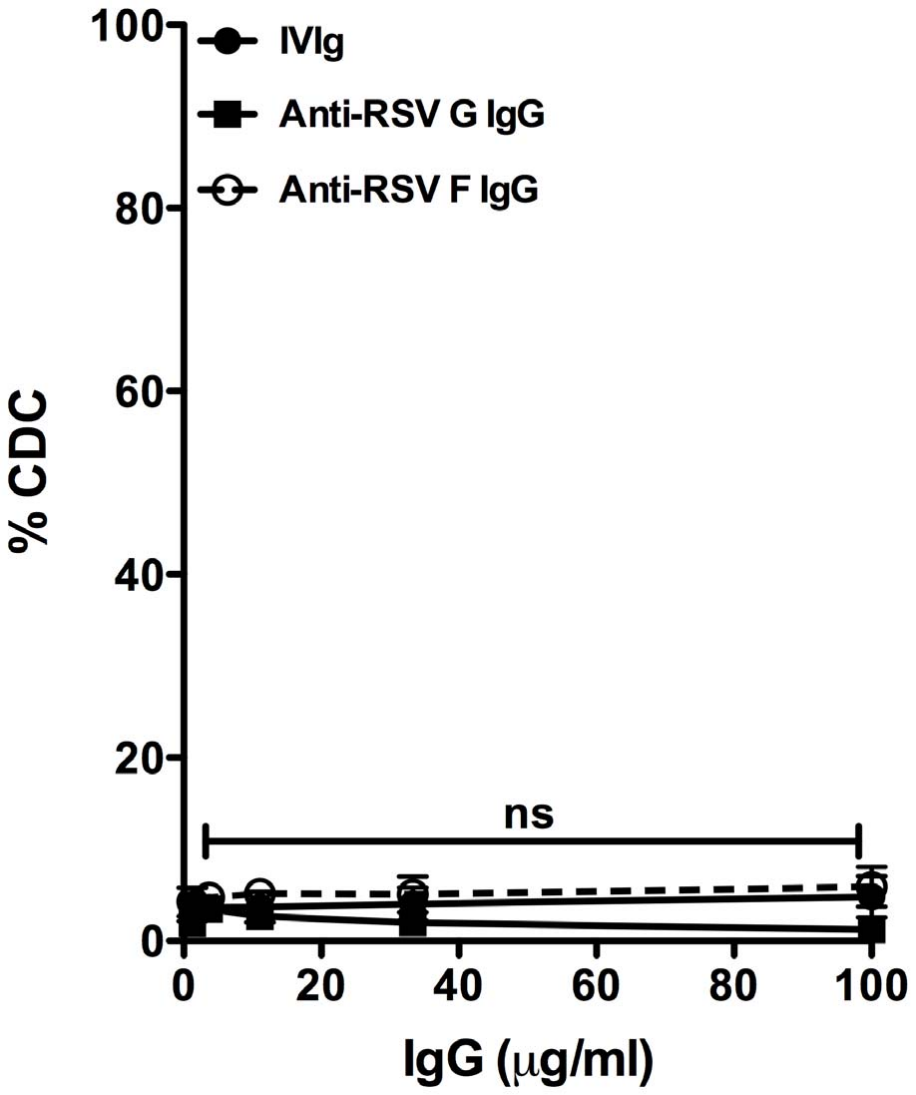
Complement-dependent cytotoxicity (CDC) activity. The RSV infected Hep-2 cells were used, as target cells and rabbit complement serum was the source of complement. The cells were incubated with various concentrations of IVIg (line with circle), anti-RSV G IgG (line with square) and anti-RSV F IgG (line with blank circle), in triplicates. Data were analyzed by two-way ANOVA followed by Bonferroni test for comparison between IVIg, anti-RSV G IgG and anti-RSV F IgG treated cells. Results are mean ± standard error of values obtained in three independent experiments. ns: non-significant.

To extend our observations regarding the functional potency of these antibodies we further analyzed their efficiency for complement-dependent cytotoxicity of HEp-2 cells infected with RSV. These antibodies were unable, however, to mediate efficient complement-dependent lysis of RSV-infected HEp-2 cells (Figure 3). The absence of complement-dependent cytotoxicity could be associated with the manufacturing process of IVIG preparations [23].

### Conclusion

This study clearly demonstrates that in addition to previously identified pathogen-specific antibodies; IVIg also contains antibodies against RSV. The presence of anti-RSV antibodies in IVIg is undoubtedly related to the wide spread prevalence of the virus across the globe. Functionally potent RSV-specific antibodies can be concentrated from IVIg by using affinity chromatography. These concentrated antibodies display higher functional potency than native preparations. Mixed types of infections are common in children infected with RSV [24]. MAbs that are highly specific for RSV cannot control mixed infections in susceptible patients. In this context, IVIg has an advantage as it contains antibodies to large number of viral and bacterial pathogens including RSV and can provide broad-spectrum protective shield to RSV-infected patients. This information can be utilized to artificially enrich immunoglobulin preparations for the prophylaxis of mixed infections. However, the low yield of affinity-purified antibodies may limit the preparation process of such specialized products. This can be overcome by enriching the IVIg preparations by addition of monoclonal anti-RSV G MAbs with defined effector functions. The limitation of our study is not being able to extend our observation in the PBMCs from pediatric subjects because of the ethical issues in obtaining the blood samples. It is likely that pediatric subjects and adults may show variation in the magnitude of ADCC. However, it has been illustrated that ADCC by neonatal cells is comparable to that of adults [25] and can be further enhanced by pharmacologic means [25], [26]. Taken together, we believe that this report provides the basis for future studies on immunoglobulin prophylaxis against mixed infections.
